# Spinal cord stimulation in severe pharmacoresistant restless legs syndrome—two case reports

**DOI:** 10.3389/fneur.2023.1219881

**Published:** 2023-11-30

**Authors:** Sandra Hackethal, Paolo Maino, Eva Koetsier, Mauro Manconi

**Affiliations:** ^1^Sleep Medicine Unit, Neurocenter of Southern Switzerland, Lugano, Switzerland; ^2^Pain Management Center, Neurocenter of Southern Switzerland, Lugano, Switzerland; ^3^Faculty of Biomedical Sciences, Università Della Svizzera Italiana, Lugano, Switzerland; ^4^Department of Neurology, University Hospital, Inselspital, Bern, Switzerland

**Keywords:** restless legs syndrome, augmentation, epidural spinal cord stimulation, case report, periodic limb movements during sleep

## Abstract

Restless legs syndrome is a prevalent, sleep-related sensorimotor disorder with relevant impact on the patients’ quality of life. For patients suffering from severe, pharmacoresistant restless legs syndrome, few therapeutic options remain to alleviate symptoms. In this case series, two patients with severe, pharmacoresistant restless legs syndrome were treated with epidural spinal cord stimulation and repeatedly assessed with polysomnography, including sleep structure and periodic limb movements as objective biomarkers not subject to placebo effects, during a 6-month follow-up period. One of the patients experienced excellent short- and long-term efficacy on subjective symptom severity (International RLS Study group rating scale 1 vs. 34 points at 3 months) and objective sleep parameters such as sleep architecture and periodic limb movements during sleep, while the second patient only reported short-term benefits from spinal cord stimulation. Ultimately, both patients opted for removal of the device for inefficacy. Based on the complex pathophysiology of restless legs syndrome and presumed mechanism of action of spinal cord stimulation in chronic pain disorders, we provide a detailed hypothesis on the possible modulating effect of spinal cord stimulation on the key symptoms of restless legs syndrome. Apart from describing a new therapeutic option for pharmacoresistant restless legs syndrome, our findings might also provide further insights into the pathophysiology of the syndrome.

## 1 Introduction

Restless legs syndrome (RLS) is a sleep-related sensorimotor disorder with an estimated prevalence of 5% in the adult population (1). The core clinical feature of RLS is a sensory discomfort ranging from disagreeable sensation to pain, associated with an urge to move, mainly occurring in the lower limbs. To satisfy diagnostic criteria, sensory symptoms have to occur or worsen during rest in the evening or during the night and disappear or improve with movement (2). Sleep onset insomnia, periodic limb movements during sleep (PLMS), and depression often accompany RLS and lead to a substantial worsening of the patients’ quality of life (3).

Most patients show a rapid, satisfactory initial response to low-dose dopamine agonists (DAs). However, around 70% become refractory to treatment over time and might develop a serious drug-related paradoxical effect named “augmentation,” characterized by a severe worsening and anatomical spreading of symptoms, anticipation of symptom onset during the day, and worsening of symptoms induced by an increase of DA dosages (4). Introduction of opioids or different combinations of off-label medication is often required to achieve symptom control in these cases (5).

### 1.1 Cases of SCS in RLS

Few cases have been reported, describing an improvement of co-morbid RLS as an incidental finding in patients treated with spinal cord stimulation (SCS) for chronic neuropathic pain (6–8, see Table 1 for an overview).

**Table 1:** Summary table of the available literature on RLS-cases treated with SCS (spinal cord stimulation).

Author, of publication	Age (years), sex	Indication for SCS	SCS placement	Therapy	Follow-up (months)	Augmentation	IRLS baseline	IRLS follow-up
Holland et al. (6)	75, M	Back pain	T10–11	Ropinirole	24	*NA*	33	0
Byrne et al. (7)								
*Case 1*	70, F	Back pain	T5–6	*NA*	2	*NA*	23	14
*Case 2*	84, F	Back pain	T9–10	Pramipexole, topiramate, capsaicin, tramadol, and gabapentin	4	*NA*	30	18
*Case 3*	58, M	Back pain	T9–10	Gabapentin, meloxicam, topic lidocaine, and cyclobenzaprine	9	*NA*	31	2
De Vloo et al. (8)	24, M	RLS	T7–9	Zolpidem, clonazepam	33	YES	*NA*	*NA (effective)*
Adil et al. (9)								
*Case 1*	34, M	Back pain	T10–11	Oxycodone, gabapentin, and diazepam	40	*NA*	*NA*	*NA* (*effective*)
*Case 2*	54, M	Back pain	T7–8	*NA*	2	*NA*	*NA*	*NA* (*effective*)
*Case 3*	42, M	Back pain	T7–8	Pregabalin	28	*NA*	*NA*	*NA* (*effective*)
Total (*Mean number*)	6 M, 2F	7 Back Pain	T5 (1)^*^		17.7	1	29.2	8.5
			T6 (1)					
			T7 (3)					
			T8 (3)					
	55.1 (age)	1 RLS	T9 (3)					
			T10 (4)					
			T11 (2)					

IRLSS, International restless legs syndrome scale; T, thoracic; NA, Not available. ^*^The number in brackets signifies the number of times that this specific spinal segment was selected for stimulation.

The earliest report describes the case of a 75-year-old male patient with chronic lower back pain and severe RLS, who experienced a complete cessation of RLS symptoms 6 weeks after implantation of a spinal cord stimulator (6). The treatment effect remained stable during the 2-year follow-up.

Byrne et al. (7) describe three patients who received an epidural SCS for lumbar and neuropathic leg pain (Failed Back Surgery Syndrome, FBSS), who also suffered from co-morbid RLS. One patient experienced a drastic decrease of symptom severity, while the other two only experienced a mild/moderate improvement during the 3–9 months follow-up period.

Adil et al. (9) report other three similar cases of lower-back and lower extremity pain treated with SCS showing an almost complete disappearance of concomitant RLS, with a follow-up ranging from 2 to 40 months. In the same year, De Vloo et al. (8) describe the first case of SCS implanted in a young patient with the primary aim to control a severe, refractory form of RLS, with an overall good clinical outcome.

While all the above cases demonstrate a good efficacy of SCS on self-rated RLS symptoms, objective, polysomnographic data on sleep structure and PLMS are not reported.

Data from systematic reviews and meta-analyses suggest large, clinically relevant placebo effects on self-rated outcomes with, however, small to absent placebo effects on objective parameters, especially PLMS (10).

### 1.2 Patient characteristics

#### 1.2.1 Patient 1

A 44-year-old Caucasian woman, affected by migraine and fibromyalgia, was referred to our center for severe, primary RLS with augmentation, refractory to standard treatment. Diverse pharmacological regimen including either single or combination therapy with pramipexole extended release (ER, max. 1.5 mg/day), ropinirole (up to 6 mg/day), levodopa (max. 250 mg/day), gabapentin (max. 600 mg/day), pregabalin (max. 150 mg/day), oxycodone/naloxone (10/5 mg/day), and clonazepam (1 mg/day), often beneficial in the first period of administration, failed to show an enduring efficacy [International RLS Study group rating scale (IRLS) 31–36 points, very severe].

**Figure 1:**
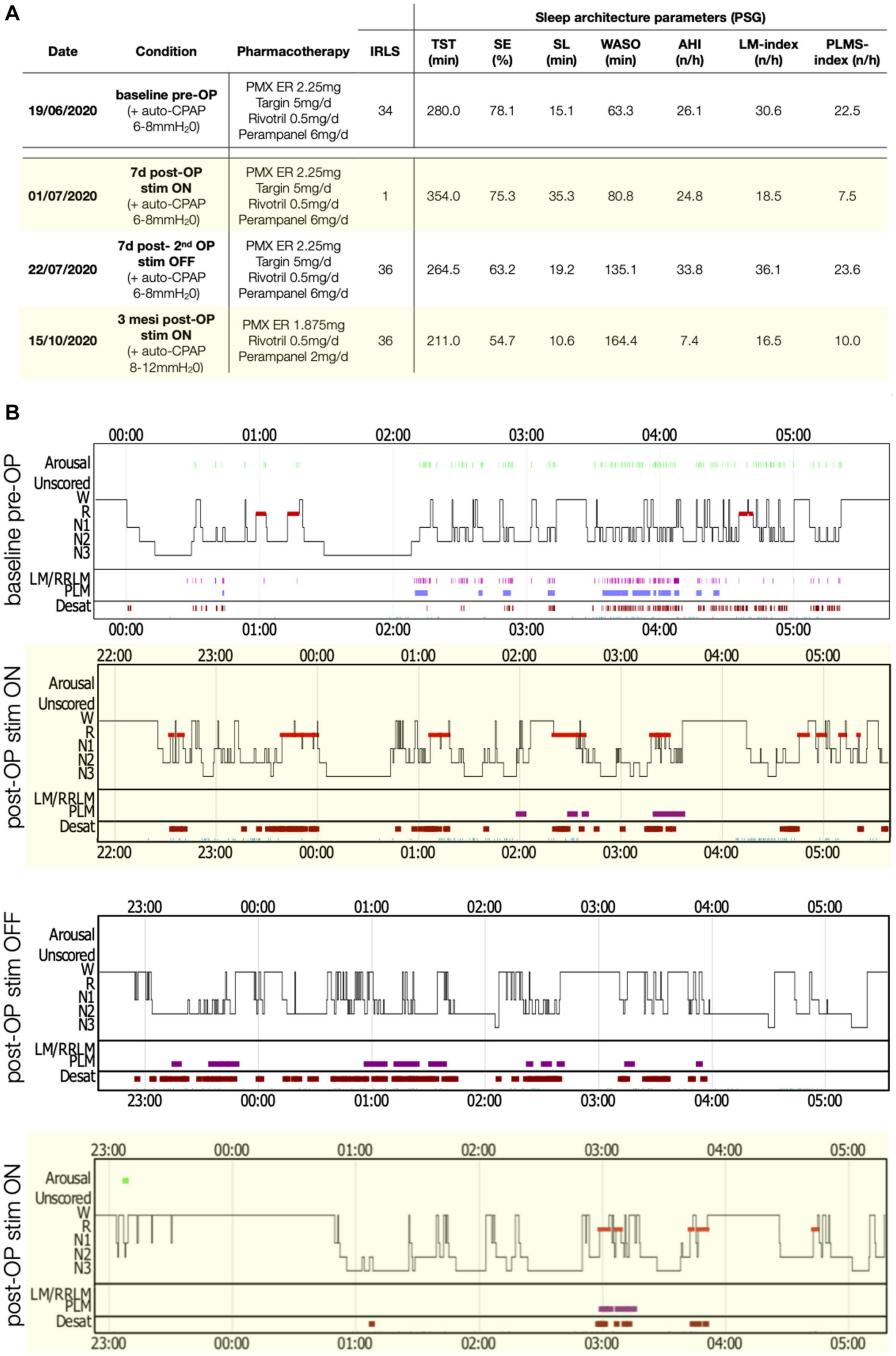
PSG parameters and sleep structure of patient 2 in the various recording conditions. **(A)** Table with sleep parameters assessed by PSG. AHI, Apnea-hypopnea index; AI, Arousal-index; C-PAP, Continuous positive airway pressure; LM, Limb movements; IRLS, International RLS Study group rating scale; PLMS, Periodic limb movements during sleep; PMX ER, Pramipexol prolonged release; SE, Sleep efficiency; SL, Sleep latency; Stim, Electro-stimulation; TST, Total sleep time; WASO, Wake after sleep-onset. **(B)** Comparisons of sleep structure between the different recording conditions. Desat, Blood oxygen level desaturation ≥4%; LM/RRLM, Leg movements/respiratory-related leg movements (also indicated as violet rectangles and bars); N1, N1 sleep; N2, N2 sleep; N3, N3 sleep; R, REM-sleep; and W, Wake.

Her baseline in-lab polysomnography (PSG) showed a severe sleep disruption [Total sleep time (TST) 347 min, Sleep efficiency (SE) 67%], a highly pathologic PLMS index (PLMSI) of 154 (number of PLMS per hour of sleep, normal value ≤15) and a normal breathing pattern (Figure 1).

#### 1.2.2 Patient 2

A 78-year-old Caucasian woman affected by ischemic heart disease, metabolic syndrome, obstructive sleep apnea syndrome (OSAS), and distal symmetric axonal polyneuropathy, was also referred to our center because of severe pharmacoresistant RLS with augmentation.

Her past pharmacological treatments included levodopa (dosage unknown), pramipexole ER (max. 2.25 mg/day), pregabalin 75 mg/day, perampanel (max. 6 mg/day) (11), oxycodone/naloxone (max. 5/2.5 mg/day), and clonazepam (2 mg/day) in different combinations, without achieving a significant long-term improvement (IRLS 30–34 points).

**Figure 2:**
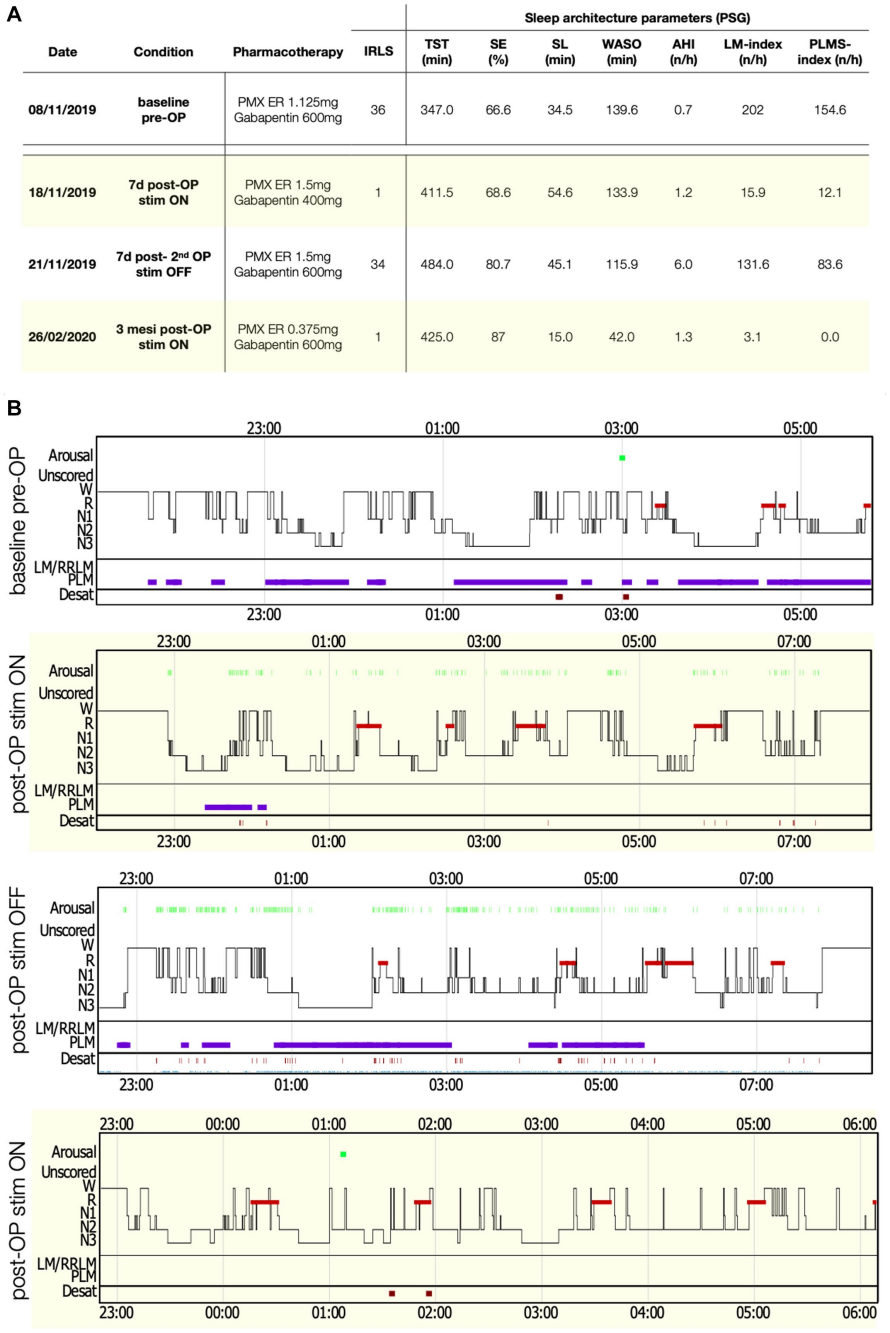
Polysomnography (PSG) parameters and sleep structure of patient 1 in the various recording conditions. **(A)** Table with sleep parameters assessed by PSG. AHI, Apnea-hypopnea index; AI, Arousal-index; LM, Limb movements; IRLS, International RLS Study group rating scale; PLMS, Periodic limb movements during sleep; PMX ER, Pramipexol prolonged release; SE, Sleep efficiency; SL, Sleep latency; Stim, Electro-stimulation; TST, Total sleep time; and WASO, Wake after sleep-onset. **(B)** Comparisons of sleep structure between the different recording conditions. Desat, Blood oxygen level desaturation ≥4%; LM/RRLM, Leg movements/respiratory-related leg movements (also indicated as violet rectangles and bars); N1, N1 sleep; N2, N2 sleep; N3, N3 sleep; R, REM-sleep; and W, wake.

Her latest PSG, using continuous positive airway pressure (C-PAP) treatment [see Figure 2 for details on ventilation parameters and residual apnea-hypopnea-index (AHI)], showed a markedly disrupted sleep (Figure 2, TST 280 min, SE 78%) and a PLMSI of 22.5.

### 1.3 Surgical intervention

**Figure 3:**
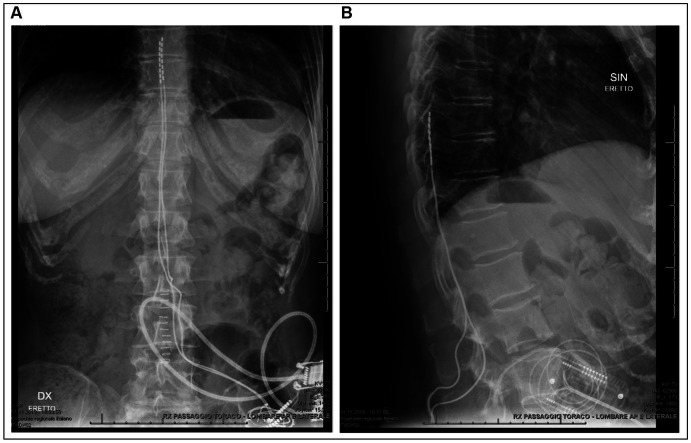
Radiography of patient 1 after definitive implantation confirming the midline position and spinal level of the lead electrodes. **(A)** Anteroposterior chest and abdominal radiography. **(B)** Lateral radiography of the thoracic and lumbar spinal column.

After multidisciplinary discussion, we proposed the two patients a trial with SCS (Spectra Wavewriter, Boston Scientific) as an experimental treatment for RLS. The patients were prepared for SCS lead implantation according to our standard pain clinic’s practice, including antibiotic prophylaxis, prone position and monitored anesthesia care. Two lead electrodes were implanted left and right to the midline in the epidural space, spanning the vertebral levels Th8-Th10, using intraoperative monitoring and patient feedback to optimize the lead position and verify parestesia coverage of the regions affected by RLS symptoms before lead fixation (see Figure 3). For both patients, a combination of tonic (supra-perception) and microburst (sub-perception) stimulation modalities was delivered simultaneously.

## 2 Results

In the first patient, SCS was immediately effective, with a complete recovery of sensory symptoms (IRLS 1 point) since the first day of administration. A PSG performed under active stimulation and stable pharmacological therapy showed a normalization of the PLMSI to 12/h (Figure 1). To confirm the benefit of SCS, another PSG without stimulation (stimulator switched off for 48 h) was performed, documenting a rebound of the PLMSI to 83/h along with an immediate relapse of RLS-symptoms (IRLS 34 points) and sleep disruption. At 3 months after SCS implantation, RLS was still controlled (IRLS 1 point), and a last PSG showed a SE of 86% together with an abolishment of PLMS (PLMSI 1/h). At 6 months after surgery, RLS symptoms gradually relapsed until SCS became ineffective and the stimulator was removed after 18 months from the implantation.

Spinal cord stimulation was also initially highly effective in the second case, with a complete recovery of sensory symptoms (IRLS 1 point). A PSG during stimulation and the same treatment as the baseline recording (Figure 2) showed a normalization of the PLMSI (7.5/h) and improvement of sleep architecture (TST 354 min). A third PSG performed after deactivation of the stimulator for 48 h again showed severe sleep disruption and a PLMSI of 23/h, with severe relapse of sensory symptoms (IRLS 34 points).

Unfortunately, after implantation of the definitive battery, the patient did not experience any benefit of the treatment, despite identical stimulation parameters used in the trial period and unchanged pharmacotherapy. Also, repeated adaptation of the stimulation parameters failed to achieve the previous level of effectiveness on the sensory symptoms, ultimately leading to the decision to remove the device after 1 year from the surgery.

## 3 Discussion

### 3.1 Hypothesized mechanism of action of SCS on RLS-symptom dimensions

The RLS symptom complex consists of three different clinical components: sensory symptoms, sleep disruption, and PLMS. From clinical evidence, drugs like DAs work better on sensory symptoms and PLMS, while alpha-2-delta (α2δ) ligands primarily act on sensory symptoms and sleep (4). This points to a complex pathophysiology, probably involving multiple neurotransmitter systems and neuronal circuits. Available evidence from neurophysiological and functional magnetic resonance imaging (MRI) studies suggests a more extensive central nervous system involvement at the spinal, cortical and subcortical level, including abnormal patterns of cortical plasticity in RLS patients, in the sense of a global “network disorder” (12, 13).

#### 3.1.1 Sensory symptoms

The sensory component of RLS spans a wide range from “urge to move” to “pain” (up to 56%), with varying degrees of uncomfortable/unpleasant sensations in between (14). Indeed, several lines of evidence imply a pathogenetic relationship between RLS and pain:

1. High comorbidity between RLS and chronic pain disorders (15).
2. Abnormalities in sensory processing are reported in both disorders, including hyperalgesia in RLS patients, which is reversible by Das (16).
3. The efficacy of opioids and α2δ ligands, with the latter reducing excitatory neurotransmitter release and modulating descending inhibition (17).
4. A hyperexcitatory state of the cerebral cortex, the thalamus, and the spinal-cord, as evident from functional MRI and electrophysiological studies are found in both pathologies (15, 18).

**Figure 4:**
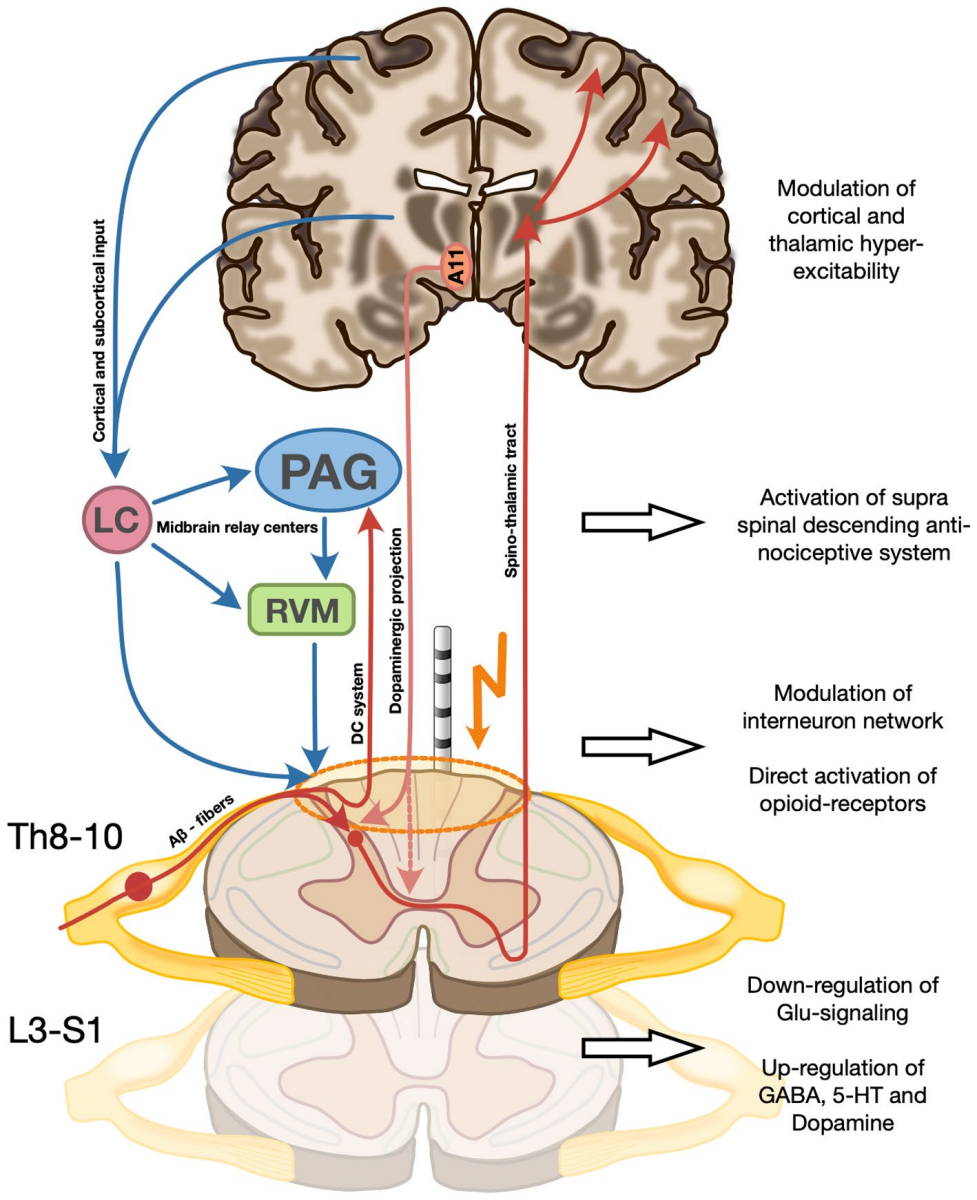
Proposed mechanism of action of SCS on the three RLS symptom dimensions on the spinal and supra-spinal level. **(A)** Direct activation of Aβ sensory fibers as well as inhibitory interneurons in the dorsal horn which exert a gamma-Aminobutyric acid (GABA)-mediated inhibition of wide-dynamic-range neurons and postsynaptic projection neurons carrying peripheral nociceptive signals in the ascending spinothalamic tract. SCS also modulates the activity of cortical and subcortical structures (incl. the basal ganglia and the amygdala), potentially modifying a more general hyperexcitatory network state incl. impaired cortical plasticity described in RLS. **(B)** Activation of dorsal column axons projecting to the periaqueductal gray, the rostral ventromedial medulla, and locus coeruleus in the midbrain, activate supraspinal inhibitory loops known as the serotoninergic and noradrenergic descending antinociceptive system (DAS). **(C)** Modulation of the sensory input and restoration of hyperexcitatory local neuronal networks to normal functional levels by SCS might lead to a reduction of local network activity to physiologic levels. Moreover, a direct frequency-dependent activation of opioid receptor subclasses has been described. **(D)** Increase of segmental dopamine levels via a possible a direct stimulation by SCS of the A11 fibers, mostly located in the dorsolateral funiculus, and thereby causing a dopamine release from the axonal terminals not only directly at stimulation level, but also at caudal levels implicated in motor control of PLM-involved muscle groups. The recruitment of the descending pathways of the DAS (see **B**) is thought to be, at least in part, the cause of increased spinal 5-HT and GABA and decreased spinal glutamate. A11, Hypothalamic dopaminergic cell group A11; LC, Locus coeruleus; PAG, Periaqueductal gray; and RVM, Rostral ventromedial medulla.

The efficacy of SCS on the sensory symptoms might mirror the same mechanism of action proposed in chronic pain. At the spinal segmental level, SCS is believed to directly activate Aβ sensory fibers as well as inhibitory interneurons located in the dorsal horn, which in turn exert a gamma-Aminobutyric acid (GABA)-mediated inhibition of wide-dynamic-range neurons and postsynaptic projection neurons carrying peripheral nociceptive signals in the ascending spinothalamic tract. Moreover, a local depolarization of dorsal column axons projecting to the periaqueductal gray, the rostral ventromedial medulla, and locus coeruleus in the midbrain, activate supraspinal inhibitory loops known as the serotoninergic and noradrenergic descending antinociceptive system (DAS, Figure 4) (19). Direct frequency-dependent activation of opioid receptor subclasses has also been shown but seems to be only a transitory effect (20).

#### 3.1.2 Periodic limb movements during sleep

Periodic limb movements during sleep occur in about 90% of patients with RLS and are very likely generated at the spinal level, driven by a local central pattern generator (CPG). The spinal interneuron network, connecting converging multimodal sensory input with the motoneuron system, is responsible for the generation of motor patterns ranging from simple reflex responses to complex motor activity including rudimental locomotion (21). The muscle recruitment observed in PLMS closely resembles the movement pattern of the propriospinal flexor reflex, pointing to a common generator of those motor phenomena (22). Furthermore, the presence of PLMS in patients with complete spinal cord transection, as well as their complete abolishment by DAs, also implicates the spinal cord as the most likely location of the CPG of PLMS and site of action of DAs (23).

Under physiological conditions, the spinal reflexogenic network activity is largely downregulated via supraspinal descending inhibitory pathways (24). During sleep, however, the descending inhibition is reduced, potentially leading to overactivity in the interneuron network facilitating the rhythmic pattern generation of PLMs.

Modulation of the sensory input as well as restoration of hyperexcitatory local neuronal networks to normal functional levels by SCS might lead to a reduction of local network activity to physiologic levels. In addition, anterograde activation of supraspinal centers in the midbrain likely contributes to reinstating inhibitory loops including the DAS, further modulating neuronal signaling in the dorsal horns (Figure 4).

With the most effective pharmacologic treatment of PLMS being D3 selective dopamine agonists, one reasonable hypothesis would be an increase of local dopamine concentration induced by SCS (5). With scarce evidence of local synthesis, the main input of dopamine is derived from the hypothalamic A11 cell group with its axons synapsing mainly on neurons of the ventral and dorsal horn of the spinal cord (25). One possibility would be a direct stimulation by SCS of the A11 fibers, mostly located in the dorsolateral funiculus, and thereby causing a dopamine release from the axonal terminals not only directly at stimulation level, but also at caudal levels implicated in motor control of PLM-involved muscle groups (Figure 4) (22, 26). A retrograde modulation of the A11 cell group at the hypothalamic level cannot be excluded.

#### 3.1.3 Sleep disruption and network dysfunction

The RLS-associated sleep disruption is characterized by reduced TST and increased SL, together with an increment of cortical arousals, which scarcely respond to DAs (27). Allen et al. (27) demonstrated a significant relationship between increased thalamic glutamatergic activity and measures of sleep disruption in RLS patients compared to controls. Moreover, α2δ ligands are thought to act mainly via decreased excitatory neurotransmitter release, such as glutamate, and thus in turn modulate a hyperexcitatory network state on the central and spinal levels (28). Modulation of thalamic activity via the ascending sensitive network, comprised of the dorsal column system and the spinothalamic tract, both of which are either directly (dorsal column system) or indirectly (by affecting the dorsal horn) acting as a relay station of peripheral sensitive information (Figure 4), could be a potential mechanism of action of SCS on RLS. Moreover, there is preclinical fMRI evidence that some forms of SCS also modulate the activity of cortical and subcortical structures including the basal ganglia and the amygdala, which have been recently implicated in the pathophysiology of RLS. A modification of the more general hyperexcitatory network state incl. impaired cortical plasticity, in line with the well-established mechanism of action of repetitive transcranial magnetic stimulation, another effective treatment option for RLS, could therefore also be hypothesized (12, 20, 29).

### 3.2 Long-term data for SCS and possible mechanisms behind loss of efficacy

Data from longitudinal trials in patients with chronic back and/or leg pain and FBSS suggest rather stable long-term efficacy of SCS with percentages of responders (defined as ≥50% pain reduction) at 6–24 months of follow-up reported ranging from 48 to 78.7% (30).

As detailed above, our second patient did not experience sustained symptom relief despite a complete symptom regression during the trial period. As of now, the reasons for this setback remain unclear, as a lead migration or dislocation was excluded. A possible explanation could be the transitory nature of direct opioid receptor activation by SCS and a tolerance to endogenous opioids, creating a ceiling effect regarding opioidergic pathway recruitment by SCS (20). Moreover, since both patients presented augmentation, whose exact pathophysiological mechanism remains only partially understood, we cannot exclude the presence of a potential non-reversable state of systemic network dysfunction associated with this complication, interfering with the general network modulating properties of SCS (see section 3.1.3).

## 4 Conclusion

Restless legs syndrome (RLS) is a frequent and disabling sleep-related movement disorder. Severe and pharmacorestiant cases of RLS represent one of the most complex therapeutic challenges nowadays in sleep medicine, especially after the observation of growing cases of *augmentation*. In this report, we describe the effect of epidural spinal cord stimulation in two cases of severe refractory RLS. The excellent short-term efficacy on all three symptom dimensions as documented by systematic longitudinal assessment of the effect of spinal stimulation on polysomnographic data, including sleep structure and periodic limb movements as objective biomarkers not subject to placebo effects, suggests SCS to be a promising new treatment option for otherwise treatment resistant RLS. Moreover, our findings might provide new insights about the pathophysiologic mechanisms behind the syndrome. However, the exact cause of the observed loss of efficacy during follow-up remains to be elucidated. Future systematic assessment in larger patient cohorts, with and without augmentation, will be necessary to better clarify long-term efficacy and establish defined inclusion criteria of patients likely to benefit from this treatment.

## Data availability statement

The raw data supporting the conclusions of this article will be made available by the authors, without undue reservation.

## Ethics statement

Ethical review and approval was not required for the study on human participants in accordance with the local legislation and institutional requirements. The participants provided their written informed consent to participate in this study. Written informed consent was obtained from the individual(s) for the publication of any potentially identifiable images or data included in this article.

## Author contributions

SH and MM analyzed data and wrote the main manuscript text. SH, PM, EK, and MM followed up both patients, collected data, and read and approved the final revision of the manuscript. All authors contributed to the article and approved the submitted version.
